# β-Cells with Relative Low HIMP1 Overexpression Levels in a Transgenic Mouse Line Enhance Basal Insulin Production and Hypoxia/Hypoglycemia Tolerance

**DOI:** 10.1371/journal.pone.0034126

**Published:** 2012-03-21

**Authors:** Xiaoping Zhang, Linda Degenstein, Yun Cao, Jeffrey Stein, Kwame Osei, Jie Wang

**Affiliations:** 1 Department of Internal Medicine, The Ohio State University, Columbus, Ohio, United States of America; 2 Transgenic Core, The University of Chicago, Chicago, Illinois, United States of America; 3 Department of Medicine, The University of Chicago, Chicago, Illinois, United States of America; University of Bremen, Germany

## Abstract

Rodent pancreatic β-cells that naturally lack hypoglycemia/hypoxia inducible mitochondrial protein 1 (HIMP1) are susceptible to hypoglycemia and hypoxia influences. A linkage between the hypoglycemia/hypoxia susceptibility and the lack of HIMP1 is suggested in a recent study using transformed β-cells lines. To further illuminate this linkage, we applied mouse insulin 1 gene promoter (MIP) to control HIMP1-a isoform cDNA and have generated three lines (L1 to L3) of heterozygous HIMP1 transgenic (Tg) mice by breeding of three founders with C57BL/6J mice. In HIMP1-Tg mice/islets, we performed quantitative polymerase chain reaction (PCR), immunoblot, histology, and physiology studies to investigate HIMP1 overexpression and its link to β-cell function/survival and body glucose homeostasis. We found that the HIMP1 level increased steadily in β-cells of L1 to L3 heterozygous HIMP1-Tg mice. HIMP1 overexpression at relatively lower levels in L1 heterozygotes results in a negligible decline in blood glucose concentrations and an insignificant elevation in blood insulin levels, while HIMP1 overexpression at higher levels are toxic, causing hyperglycemia in L2/3 heterozygotes. Follow-up studies in 5–30-week-old L1 heterozygous mice/islets found that HIMP1 overexpression at relatively lower levels in β-cells has enhanced basal insulin biosynthesis, basal insulin secretion, and tolerances to low oxygen/glucose influences. The findings enforced the linkage between the hypoglycemia/hypoxia susceptibility and the lack of HIMP1 in β-cells, and show a potential value of HIMP1 overexpression at relatively lower levels in modulating β-cell function and survival.

## Introduction

Pancreatic β-cell failure in diabetes is characterized primarily by progressive loss of insulin production and β-cell mass. β-cell failure has been attributed to autoimmune assault in type 1 diabetes and to glucolipotoxicity, amyloid deposition, insulin resistance, and endoplasmic reticulum (ER) and/or oxidative stress in type 2 diabetes [1]–[7]. However, the intrinsic mechanisms underlying β-cell susceptibility to stress and damage remains largely unclear. Are β-cells overwhelmed by such stress and its associated toxic byproducts, such as reactive oxygen species (ROS), or does the attenuation of beneficial genes and/or trigger of harmful genes prove detrimental to some inherent property specific to cell type? Data exist to support both views, but β-cell dysfunction and damage resulting from various stress insults, such as from hypoglycemia/hypoxia [8], [9] and chronic hyperglycemic and hyperlipidemic conditions [7], [10]–[12], in addition to influences by genetic variations support the second view.

For example, the lack of regular blood flows in early hours of islet transplantation leads to insufficient supplies of oxygen and nutrients (e.g., glucose) within implanted islets. This hypoglycemia/hypoxia influence is implicated in β-cell damages within implanted islets, which limits a long-term success of this technique for patients with type 1 diabetes in clinical practice [8], [13]–[16]. Likewise, as β-cells mature to produce insulin, they become sensitive to cytokine insult [17]. Insulin is the most abundant and unique protein manufactured in β-cells. Proinsulin, the predominant form of insulin precursor in the ER, preserves a low relative folding rate, and bears the greatest burden in the protein folding of β-cells. Thus, it maintains a homeostatic balance of natively and plentiful non-natively folded states (i.e., proinsulin homeostasis, PIHO) in β-cells as a result of the integration of maturation and disposal processes [18]. The low relative folding rate and plentiful insulin precursor manufactured in β-cells make PIHO susceptible to genetic and environmental influences, and PIHO disorder has been critically linked to defects in β-cells in diabetes [18].

Moreover, low expression levels of genes protective against oxidative stress, such as catalase, in the β-cells is proposed to contribute to β-cell stress susceptibilities [19], [20]. Recently, in our efforts to identify important differences in gene expression in pancreatic α- versus β-cells, a gene termed *HIMP1* (hypoglycemia/hypoxia inducible mitochondrial protein) and its protein product has been characterized in α-cells [21]. *HIMP1*, also known as hypoxia induced gene 1 (*Hig1*), is a main characterized member of a gene family conserved throughout evolution, with members in α-proteobacteria and diverse eukaryotic taxa such as fish and humans [21], [22]. HIMP1 is also called Higd-1a [23]. In mice, its two alternatively spliced products (HIMP1-a and HIMP1-b) each form a transmembrane loop, having an N_outside_–C_outside_ orientation and are expressed highly in the mitochondrial inner membrane in several tissues including heart and pancreatic α-cells, but are not or are only lowly expressed in β-cells [21]. Ectopic expression of HIMP1 in MIN6 β-cells protects the cells from apoptosis induced by hypoglycemia and hypoxia insults and prolongs their survival, suggesting an important role for HIMP1 in stress protective programs in mitochondria [21]. A recently reported study has shown that the anti-apoptotic effect of HIMP1 results from inhibiting cytochrome C release and reducing caspase activities in mouse macrophage cell lines [23].

To further understand the intrinsic mechanisms for hypoglycemia/hypoxia susceptibilities of β-cells, we produced novel transgenic (Tg) mice with HIMP1-a overexpression by the control of mouse insulin 1 gene promoter (MIP, ∼8.3 kb in length). The application of MIP allows ectopic HIMP1-a expression only within pancreatic β-cells in body. Results of our characterizations on HIMP1-Tg mice show that β-cells with relative low levels of HIMP1 have enhanced basal insulin production and tolerances to hypoxia/hypoglycemia. The findings validate the linkage between the hypoglycemia/hypoxia susceptibility and the lack of HIMP1 in mouse primary β-cells, and show a potential value of HIMP1 overexpression at relative lower levels in modulating β-cell function and survival.

## Results

### Levels of HIMP1 overexpression in β-cells determined phenotypes in HIMP1-Tg mice

**Figure 1:**
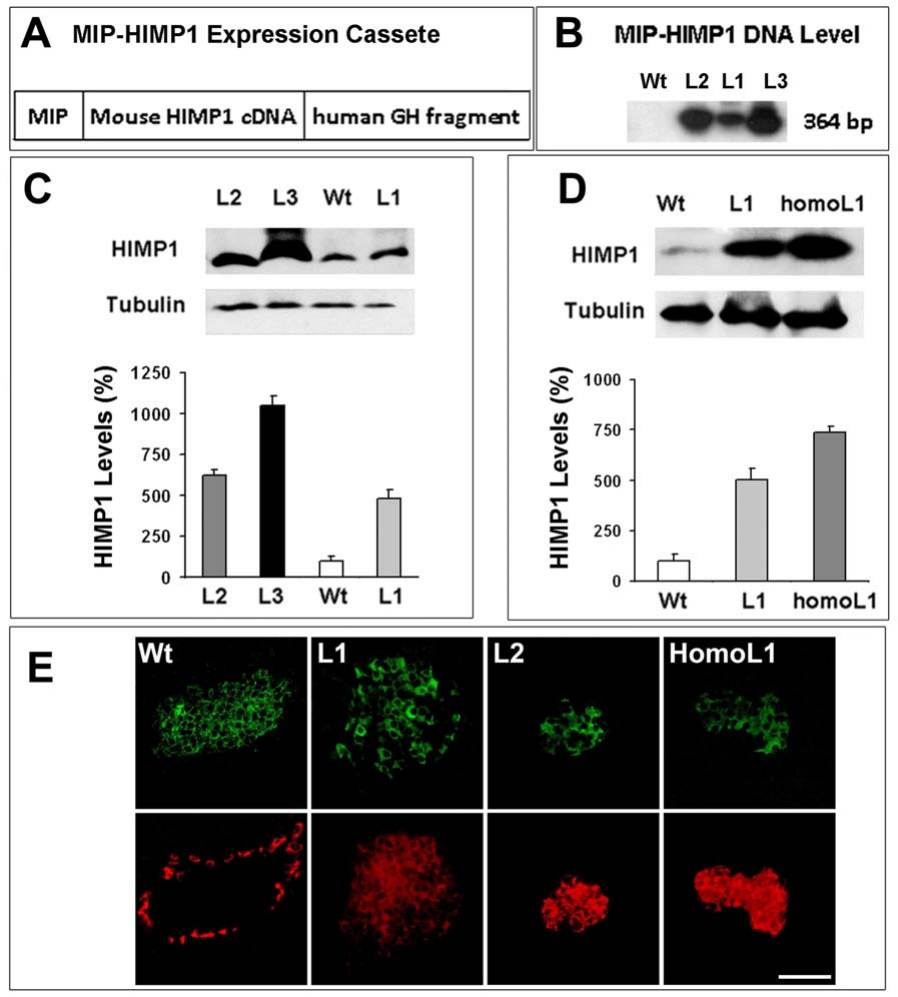
The levels of MIP-HIMP1 expression cassette DNA and HIMP1 protein in pancreatic/islet tissues of HIMP1-Tg mice. (A) Diagram of MIP-HIMP1 expression cassette. MIP, mouse insulin1 promoter, GH, a fragment of growth hormone gene. (B) The levels of MIP-HIMP1 expression cassette DNA in HIMP1-Tg mice were examined by quantitative PCR methods with ^32^P-labeled primers as described previously [24]. Pancreatic (C) or islet (D) HIMP1 protein levels of 5–30-week-old HIMP1-Tg mice were determined by immunoblot analysis, normalized by tubulin, and compared with wild-type littermate controls. (E) Representative images of HIMP1 (red) and C-peptide (green) immunoreactive materials in pancreatic tissues of 5-week-old male HIMP1-Tg and control mice. 50 µg proteins per lane in (C) and (D). Wt, wild-type littermate; L1, L1 heterozygotes; L2, L2 heterozygotes; L3, L3 heterozygotes; homoL1, L1 homozygotes. The data in (C) and (D) were shown as mean ± SD, n = 6; **, *P*<0.01. Bar in (E), 100 µm.

Three transgenic founders were identified by amplification of a DNA fragment (364 bp) specifically from the MIP-HIMP1 expression cassette (diagrammed in Fig. 1A) rather than natural HIMP1 gene in the genome of mouse as described in the Materials and Methods. Heterozygous HIMP1 transgenic mice of 3 lines denoted as ‘HIMP1-Tg L1, L2 and L3’ were produced by breeding of transgenic founders with C57BL/6J mice. Levels of the MIP-HIMP1 expression cassette DNA that was integrated in the genome of HIMP1-Tg mice were examined by using genomic DNAs of tail tissues as templates and the quantitative PCR approach described previously [24]. Results showed a steady increase of the MIP-HIMP1 expression cassette DNA level from L1 to L3 line heterozygotes. But no signal was evident in wild-type littermates because these mice do not carry the MIP-HIMP1 expression cassette DNA in their genome (Fig. 1B). Phenotype characterizations (detailed later) exposed a negligible decline in the blood glucose level of 5–30-week-old L1 heterozygous mice, while hyperglycemia appeared in 5-week-old L2/3 heterozygous mice with no evident obesity or significant sex differences. Genetic/phenotype traits of HIMP1-Tg mice followed the mode of Mendelian inheritance. The differences in the level of the MIP-HIMP1 expression cassette DNA (Fig. 1B) may result from variations in the number of MIP-HIMP1 DNA integrated in genome, which may potentially be responsible for discrepancies in the phenotype of HIMP1-Tg mice between 3 lines.

The level of HIMP1 protein in the pancreatic tissues of L1 to L3 heterozygotes increased steadily than did in wild-type littermate controls (Fig. 1C; control, 100±10%; L1, 480±21%; L2, 624±13%; L3, 1050±60%; n = 5, *P*<0.01). This result further supports that there is a linkage between the HIMP1 overexpression levels and the phenotype variations in HIMP1-Tg mice of 3 lines. To prove this linkage, we produced L1 homozygotes by breeding of L1 heterozygote siblings. As expected, hyperglycemia (11.6±0.6 mmol/l) developed by 5 weeks of age in L1 homozygotes, which preserved double the amount of MIP-HIMP1 DNA than did L1 heterozygote parents. The level of HIMP1 protein in the islets of L1 homozygotes versus wild-type controls (Fig. 1D; control, 100±22%; L1, 500±16%; L1 homozygote, 738±22%, n = 5, *P*<0.01) is comparable to the level in L2 heterozygotes (Fig. 1C). This genetic evidence enforced the linkage between the levels of HIMP1 overexpression and the phenotypes in HIMP1-Tg mice.

To elucidate whether the increase of HIMP1 protein occurs specifically in β-cells, we performed histological studies on pancreatic sections of HIMP1-Tg mice (but excluded the L3 line which was lost by accident). As shown in Fig. 1E, HIMP1 immunoreactivities were evident in α-cells rather than in islet β-cells (marked with C-peptide immunoreactivities) or exocrine cells of normal pancreatic tissues, which is consistent with our previous observation [21]. In contrast, HIMP1 immunoreactivities were clearly evident as well in the islet β-cells (marked with C-peptide immunoreactivities) of HIMP1-Tg mice. Moreover, an increased tendency in the HIMP1signals was shown in pancreatic tissues derived from L1 to L2 heterozygotes or L1 homozygotes (Fig. 1E). These results concluded that differences in the levels of HIMP1 overexpression in β-cells are responsible for the phenotype traits of HIMP1-Tg mice. The studies shown below mainly are the characterizations on consequences of HIMP1 overexpression at relatively lower levels in L1 heterozygotes. Toxic consequences of HIMP1 overexpression at higher levels in diabetic L1 homozygotes or L2 heterozygotes will be characterized and described in details elsewhere.

### Heterozygous HIMP1-Tg-L1 mice show a negligible decline in blood glucose levels

**Figure 2:**
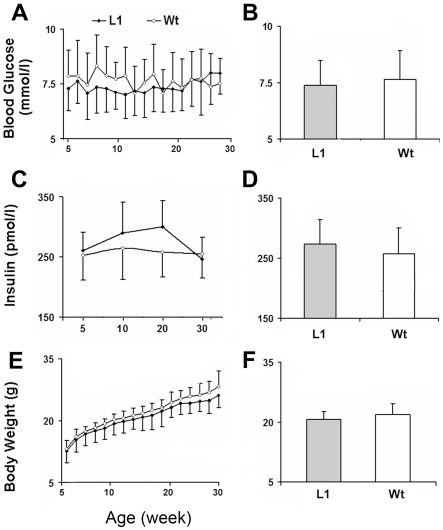
Phenotypes of heterozygous HIMP1-Tg-L1 mice. Blood glucose (A, n = 30) or insulin (C, n = 10) level and body weight (E, n = 30) of 5–30-week-old L1 heterozygotes or wild-type (Wt) littermates were measured under *ad libitum* feeding conditions and the average values were shown in B, D, and F. Equal numbers of male and female mice in individual groups were tested. Data were shown as mean ± SD.

Under *ad libitum* feeding conditions, 5–30-week-old heterozygous HIMP1-Tg-L1 mice showed, only slightly and insignificantly, lower blood glucose levels (Fig. 2A and B), less body weight (Fig. 2E and F), and higher insulin levels (Fig. 2C and D) than did wild-type littermate mice. Moreover, no apparent alteration in these phenotype traits was found in L1 heterozygotes during a long-term (two years) observation. These data indicate that heterozygous HIMP1-Tg-L1 mice preserve a negligibly improved glucose metabolism. In contrast, HIMP1 overexpression at toxic higher levels caused hyperglycemia in L1 homozygotes and L2/3 heterozygotes by 5 weeks of age (Fig. S1) as introduced early in the Results section.

### β-cells of heterozygous HIMP1-Tg-L1 mice show an enhanced tolerance to low oxygen/glucose insults

**Figure 3:**
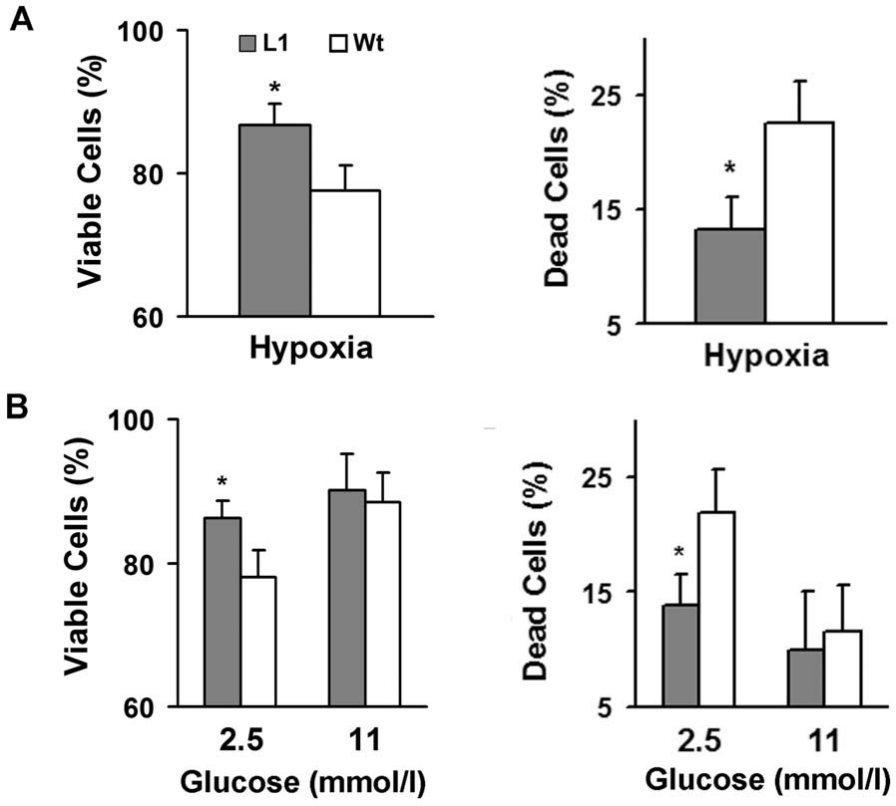
Heterozygous HIMP1-Tg-L1 islet cells show an enhanced tolerance to low oxygen or glucose insults. Following similar procedures described previously [21], primary islet cells of 5-week-old heterozygous L1 or wild-type (Wt) littermate mice were dispersed and cultured under (A) the hypoxia (5% O_2_) condition for 15 h or (B) under the low (2.5 mmol/l) or high (11 mmol/l) glucose conditions for 48 h. Viable or dead cells (via apoptotic and/or necrotic pathways) were examined by trypan blue staining, and the percentages calculated by the procedures described in the Materials and Methods are shown as mean ± SD. n = 6; *, *P*<0.05.

To examine whether primary L1 β-cells enhances tolerance to hypoxia/hypoglycemia stress as that observed in β-cell lines with transfected HIMP1 cDNAs [21], we applied similar approaches as reported [21] and examined the viability of islet cells derived from L1 mice and wild-type littermates by the method of trypan blue staining. This method can distinguish viable cells from dead cells via apoptotic or necrotic pathways. Examination results show that, after a 15 h incubation at the hypoxia (5% O_2_) or 48 h low glucose (2.5 mmol/l) condition, the proportion of viable L1 cells increased with a decreased proportion of dead L1 cells by comparison with wild-type controls (Fig. 3, *P*<0.05, n = 6). In contrast, no significant difference was found in the viability of L1 islet cells versus wild-type islet cells that were cultured at the customary glucose (11 mmol/l) condition (Fig. 3). These results showed the role of HIMP1 in protective programs of islet primary β-cells against low oxygen/glucose insults. This is because HIMP1 overexpression is the primary genetic alteration in L1 β-cells and no change in α- and β-cell populations (that is described in the last paragraph of the Result section) contributes to the increased viability of HIMP1-Tg-L1 islet cells.

### Heterozygous HIMP1-Tg-L1mice show an enhanced basal insulin secretion

**Figure 4:**
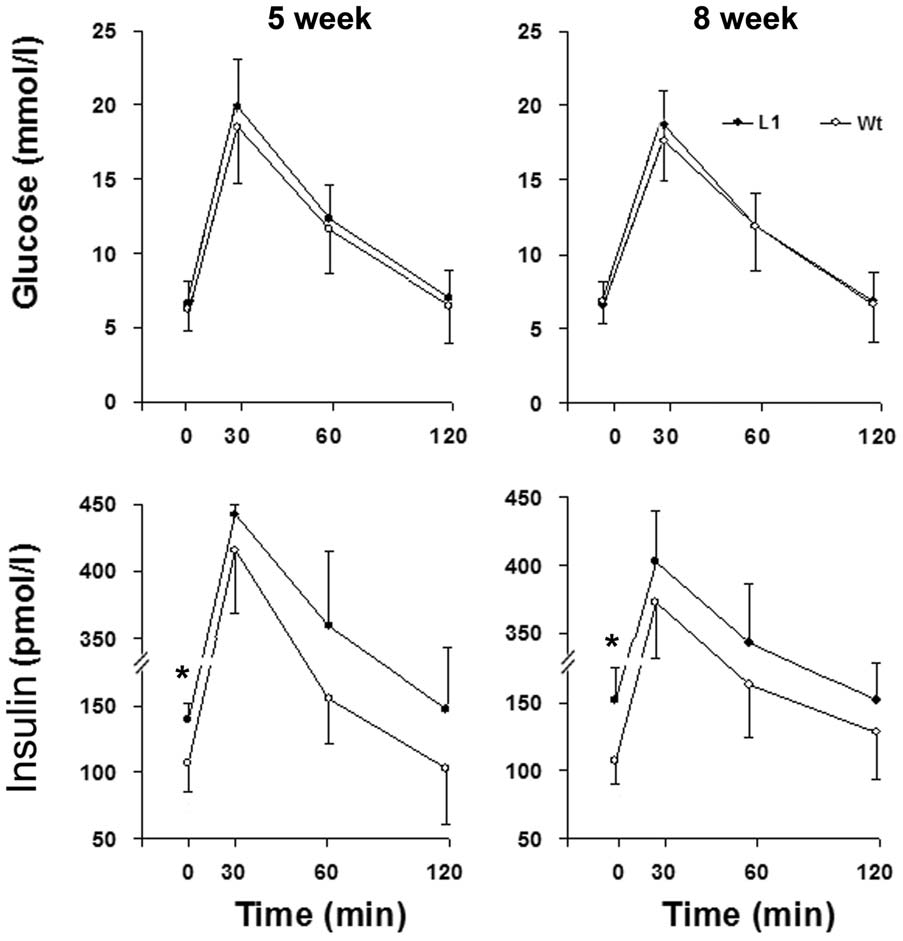
Heterozygous HIMP1-Tg-L1 mice show an enhanced basal insulin secretion. 5- and 8-week-old heterozygous L1 and wild-type (Wt) littermate mice were fasted for 15 h and then subjected to IPGTT studies (2 g/kg body weight). Equal numbers of male and female mice were tested in individual groups. The blood glucose or insulin levels measured in IPGTT studies are shown. The data in (A to D) are presented as the mean ± SD. n = 10; *, *P*<0.05.

To investigate the effect of HIMP1 overexpression on insulin secretion of L1 β-cells *in vivo*, we performed intraperitoneal glucose tolerance test (IPGTT) and examined basal (fasting) insulin release and glucose-stimulated insulin secretion (GSIS) by β-cells of 5- and 8-week-old L1 heterozygotes. Results exposed no apparent defects in GSIS or glucose tolerance in heterozygous HIMP1-Tg-L1 mice by comparison with littermate controls (Fig. 4). A similar result was found in 30-week-old L1 heterozygotes, and these observations were evident as well by analyses of AUC glucose and AUC insulin in IPGTT studies (data not shown). However, an increase in basal insulin levels were detected in 5- and 8-week-old L1 heterozygous mice after a 15 h fasting (Fig. 4, *P*<0.05; n = 10). This result indicates that HIMP1 overexpression at relatively low levels in heterozygous HIMP1-Tg-L1 β-cells has enhanced basal insulin secretion.

### Heterozygous HIMP1-Tg-L1 islets show an enhanced basal (pro)insulin biosynthesis

**Figure 5:**
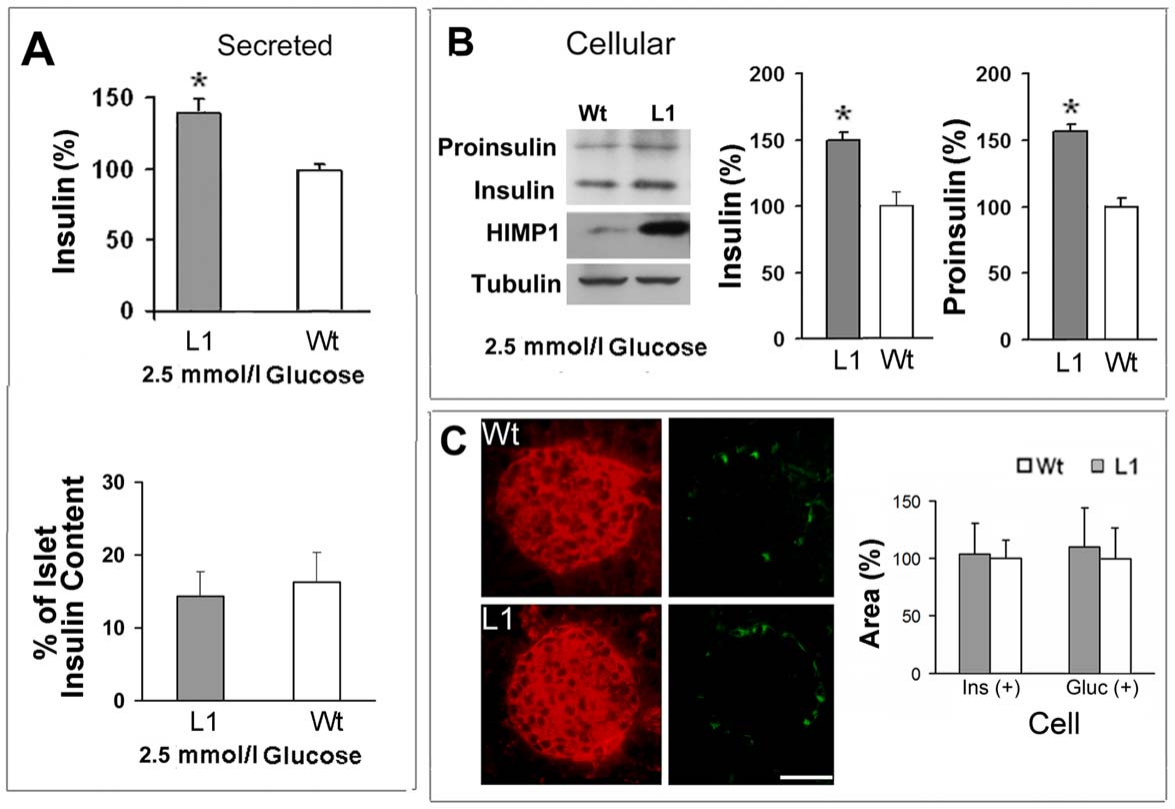
Heterozygous HIMP1-Tg-L1 islets show an enhanced basal (pro)insulin biosynthesis. Islets isolated from 5-week-old heterozygous HIMP1-Tg-L1 or wild-type (Wt) mice were cultured for 15 h at the 2.5 mmol/l glucose condition. Insulin secreted during the 15 h culture was examined by RIA, and the proportion of secreted insulin in the islet insulin content was shown in (A, lower panel). The proinsulin and insulin contents of L1 and Wt islets were determined by insulin antisera alone on the same blot membrane, normalized by tubulin, and shown in (B). (C) The α-cell (determined by glucagon immunoreactivity, green) and β-cell (determined by insulin immunoreactivity, red) area per sectional islet unit in the pancreatic tissues of 5-week-old L1 heterozygote and wild-type littermate mice was analyzed using ImageJ software. Data in (A to C) were shown as mean ± SD. n = 6; *, *P*<0.05. Scale bar in (B), 100 µm.

To investigate mechanisms for the enhanced basal insulin secretion from L1 β-cells, we performed *ex vivo* studies on 5-week-old heterozygous L1 islets. After a 15 h culture under the 2.5 mmol/l glucose condition, an increase occurred in the level of secreted insulin from L1 islets compared to wild-type controls in radioimmunoassay (RIA) studies (Fig. 5A, *P*<0.05, n = 6), despite that no difference appeared in the percentage of secreted insulin in the islet insulin content (Fig. 5A, lower panel). Using immunoblot methods described recently [18], our further examination on islet proteins revealed that an increase also appeared in the proinsulin and insulin content of L1 islets compared to wild-type controls (Fig. 5B, *P*<0.05, n = 6). Because β-cell population in L1 islets versus size-matched control islets is unaltered (Fig. 5C), these results together demonstrated that HIMP1 overexpression at relatively low levels in L1 β-cells has enhanced basal (pro)insulin biosynthesis and insulin secretion *in vivo*. Under the high glucose (11 mmol/l) customary culture condition, however, no significant difference was found in the (pro)insulin content or secreted insulin of the two islet groups, despite the slight increase that indeed did occur in L1 islets versus wild-type controls (Fig. S2). These findings are consistent with observations in the *in vivo* IPGTT studies (Fig. 4).

## Discussion

In our efforts to develop better understanding of the mechanisms for β-cell susceptibility to hypoxia/hypoglycemia, we have produced 3 lines of HIMP1-Tg mice with HIMP1 overexpression specifically within β-cells in this study. Analysis of genomic DNAs and pancreas/islet proteins uncovered a steady increase of HIMP1 level in β-cells of L1 to L3 heterozygotes (Figs. 1B, C and D). Intriguingly, phenotype characterizations exposed a slight and insignificant improvement in glucose metabolism of L1 heterozygotes that preserve relatively low levels of HIMP1 overexpression (Figs. 1 and 2). In contrast, hyperglycemia developed early in 5-weeks-old L2/3 heterozygotes that preserve relatively high levels of HIMP1 overexpression. These observations suggest that the variation in phenotype is correlated with the difference in the level of HIMP1 overexpression in the 3 lines of HIMP1-Tg mice. This proposition was proven by the fact that hyperglycemia developed in L1 homozygotes that were inherent with double amounts of MIP-HIMP1 DNAs and higher HIMP1 overexpression levels than that in heterozygote parents (Fig. 1D and E). These data also suggest that HIMP1 overexpression at relatively low levels in β-cells exerts somewhat beneficial effects on β-cell function while at higher levels would result in toxic consequences such as β-cell failure and diabetes. Because diabetes often developed in engineered animals with overexpression of some (even beneficial) molecules under the control of strong insulin promoter [25]–[28], the fact that the disease developed in L1 homozygotes and L2/3 heterozygotes due to toxic higher levels of HIMP1 overexpression is comprehensible. Additionally, HIMP-L3 mice were lost because of accidents that happened during maintenance processes.

Our studies on dispersed L1 islet cells show that HIMP1 overexpression at relatively low levels has enhanced the viability of β-cells and the tolerance to cell death induced by low oxygen/glucose insults (Fig. 3). This finding and a recently reported study in mouse macrophage cell lines [23] further validated the suggested role of HIMP1 in protective programs of cells against low glucose and hypoxia insults [21]. These data provide better understanding of function(s) of HIMP1protein and its homologues. Function(s) of these proteins as members of a novel family remains to be established.

Our IPGTT studies found a significant increase in fasting (basal) insulin levels of L1 heterozygous mice (Fig. 4). In line with this result, the outcomes of *ex vivo* islet studies further showed that, after a 15 h incubation at the 2.5 mmol/l glucose condition, there was an increase in the (pro)insulin content and secreted insulin of L1 islets versus wild-type controls (Figs. 5A and B). Because β-cell populations remained unaltered in L1 versus size-matched control islets (Fig. 5C), these data clearly demonstrated that HIMP1 overexpression at relatively low levels in heterozygous L1 β-cells has enhanced basal (pro)insulin biosynthesis and basal insulin secretion.

Previously reported studies show that overexpression of some anti-oxidative enzymes and mitochondrial proteins such as catalase or Bcl(X)L [25], [26] specifically in β-cells improved cell survival capacity but not (pro)insulin biosynthesis. This study shows the first clear evidence that β-cells with HIMP1 overexpression at relatively low levels have enhanced basal (pro)insulin production in addition to tolerances to low oxygen or glucose insults. This finding is quite intriguing because it indicates that enhancements in the basal insulin synthesis and secretion can be achieved by overexpression of only a mitochondrial inner membrane protein in β-cells. This enhancement would be incomparable with the increase in the basal insulin secretion observed in type 2 diabetes with obesity [29], [30]. This is because obese and diabetes does not develop in heterozygous HIMP1-Tg-L1 mice. Moreover, the increase in the basal insulin secretion in type 2 diabetes with obesity is associated with a decrease in the β-cell's intracellular stores that cannot be offset by commensurate free fatty acid induction of proinsulin biosynthesis [30]. In addition, the increased basal (pro)insulin synthesis in heterozygous L1 islets (Fig. 5B) suggests that transcriptional and/or translational regulations for the increase may occur somehow. However, it remains to the clarified what or how mitochondrial messages changed as a result of HIMP1 overexpression that link to regulations in the transcription, translation, and secretion processes of insulin in L1 islets/β-cells. In addition, whether there is any possible means to regulating optimal expressions of natural *HIMP1* gene in β-cells remains to be investigated. A recently reported study showed that transcription of HIMP1 is regulated by binding of the transcription factor HIF to the hypoxia-response element site in the HIMP1 promoter in mouse macrophage cell lines [23]. HIF is expressed in pancreatic β-cells as well [9], [31], [32], but why the HIMP1 protein is not normally expressed in mouse pancreatic β-cells remains unclear. There may be differences in the expression level or post-translational modifications of HIF and/or other involved transcription factors between pancreatic α- and β-cells. This possibility needs to be further clarified in follow-up studies.

In summary, our findings provide new insights into the mechanisms for susceptibilities of β-cells to hypoxia/hypoglycemia influences such as at the microenvironment within early implanted islets in transplantation. Also, our findings clearly demonstrate the sensitivity of basal insulin biosynthesis and secretion to changes in mitochondria, and show a potential value of HIMP1 overexpression at relatively low levels in modulating β-cell survival and function. In diabetes treatments, the potential value of HIMP1 expression ectopically in primary β-cells of transgenic animals may lie in islet transplantation. Expression of HIMP1, perhaps in combination with other protective molecules such as antioxidant enzymes, may be a possible approach to reduce primary β-cell nonfunction of islet grafts by using nonhuman donor animals such as transgenic pigs. In addition, to reveal and apply any possible means to regulating optimal expressions of natural *HIMP1* gene in β-cells will be helpful for protection of β-cells in diabetes treatments. The mechanisms by which HIMP1 overexpression at lower levels enhances basal insulin production and β-cell survival while at toxic higher levels causing β-cell failure and diabetes deserve further study. HIMP1-Tg mice maintaining in a number of lines provide an ideal tool for answering these questions.

## Materials and Methods

### Ethics statement

All animal and tissue sample experiments have been approved by the Institutional Animal Care and Use Committee of The Ohio State University (protocol number 2007A0040 and 2010A0024) and were performed in accordance with the guidelines of the National Institutes of Health and The Ohio State University.

### Generation of HIMP1 transgenic mice

The expression cassette of MIP with a fragment (∼2.1 kb) of human growth hormone gene for stable high-level expression in pGEM-11Zf vector (Promega, Sunnyvale, CA, USA) was used [33], [34]. Mouse HIMP1-a cDNAs (∼0.36 kb) amplified with a set of primers (5′-CTTCTCGAGGCGGCCAGAAACCGGCAGGAC-3′ and 5′-AGTCTCGAGAGCTCTTCTAAGGCTTAGGGC-3′) was inserted into the Xho1 cloning site in the MIP expression cassette and determined by Sequencing. The constructed MIP-HIMP1 expression cassette was injected into the pronuclei of fertilized oocytes from C57BL/6J mice (The Jackson Laboratory, Bar Harbor, Maine, USA). Three founders were obtained and transgenic mice of 3 lines denoted as ‘HIMP1-Tg L1, L2 and L3’ were produced by breeding of transgenic founders with C57BL/6J mice. HIMP1-Tg mice were identified by PCR using genomic DNAs obtained from mice tails as the template. Because a set of primers (5′-CAGCGATTGTTGCCTATGGGTTGT-3′ and 5′-CTGGGCTTAGATGGCGATACTCAC-3′) were selected specifically from the MIP-HIMP1 expression cassette DNA rather than natural *HIMP1* gene in the genome of mouse, a PCR product (364 bp) can be only amplified from HIMP1-Tg mice. The PCR was performed under the following conditions: 3 min at 94°C; 40 sec at 94°C and 80 sec at 68°C for 5 cycles; 40 sec at 94°C, 30 sec at 57°C, and 80 sec at 72°C for 38 cycles followed by 7 min at 72°C. Radioactive primers used were labeled with [γ-^32^P]ATP (1.11×10^14^ Bq/mmol, Perkin Elmer Life Science, Waltham, MA, USA) as described previously [24].

### Measurements of body weight and blood glucose or insulin levels

Mice maintained in a temperature-controlled room on a 12 h light-dark cycle were given free access to food and water. Body weight and blood glucose of HIMP1-Tg and littermate mice were measured once a week during 4–12 weeks, every two weeks during 14–30 weeks of age, and then measured occasionally afterwards. Blood samples for glucose and insulin assay were collected from mouse tail vein. Blood glucose or insulin concentrations were measured by One Touch Ultra meter (LifeScan, Milpitas, CA, USA) or Rat Insulin RIA kit (Millipore, Billerica, MA, USA).

### Islet isolation and culture

Islets isolation with collagenase (Sigma-Aldrich, St Louis, MO, USA) digestion and culture with 10% fetal calf serum (FCS)/RPMI 1640 media (containing 11 mmol/l glucose, Invitrogen, Carlsbad, CA, USA) were described previously [18], [21], [24]. Overnight cultured islets were then subjected to treatments/analyses.

### Examination of dead islet cells cultured at different oxygen/glucose conditions

Equal numbers of islet cells which dispersed by trypsin of 5-week-old L1 and littermate mice were cultured in 10% FBS/RPMI 1640 media (containing 11 mmol/l glucose) overnight. These cells were then subjected to 15 h hypoxia (5% O_2_) or to 48 hour low (2.5 mmol/l) and customary (11 mmol/l) glucose culture conditions. Viable or dead cells were examined by Trypan Blue (Invitrogen) staining method [35] and then counted in haemocytometer chambers. We calculated the percentage of viable or dead cells in total islet cells at the indicated time points using the formulas: viable cell (%) = (viable cell number)×100%/(dead cell number+viable cell number); dead cell (%) = (dead cell number)×100%/(dead cell number+alive cell number).

### Intraperitoneal (i.p) glucose tolerance test (IPGTT)

Mice at the age of 5 and 8 weeks were fasted overnight for 15 h and then injected i.p. with glucose (2 g/kg body weight). Blood glucose and insulin levels were determined from tail vein at 0 (before glucose administration), 30, 60 and 120 min after glucose administration.

### Immunohistochemistry and immunoblot

Double-staining with antibodies against to HIMP1 (1∶500) [21], rat C-peptide 2 (1∶500, Millipore), insulin (1∶2000, Millipore), and glucagons (1∶2000, Millipore) on pancreatic sections (5 µm thickness) was performed as described previously [21], [24]. Fluorescent images were examined with an Axiovert 200 microscope (Carl Zeiss, Oberkochen, Germany). Pancreas/islet proteins in tricine sample buffers were resolved by 16.5% tricine-SDS-PAGE for immunoblot analyses as described previously [18], [24].

### Statistical analysis

Densitometry of protein bands in immunoblot images or densitometry of fluorescent images were quantified by ImageJ software. Data are shown as mean ± SD. Statistical significance (*P<0.05, ** *P*<0.01) was assessed by two-tailed Student's *t*-test or analysis of variance if appropriate. All experiments were carried out at least three times.

## Supporting Information

Figure S1 **Blood glucose concentrations of 5-weeks-old L1 homozygotes and L2/3 heterozygotes.**
(TIF)

Figure S2 **The (pro)insulin content and secreted insulin level of HIMP1-Tg-L1 and control islets after 15 h culture at the 11 mmol/l glucose condition.** Islets isolated from 5-week-old heterozygous HIMP1-Tg-L1 or wild-type (Wt) mice were cultured for 15 h at 2.5 mmol/l glucose conditions. Insulin secreted during the 15 h culture was examined by RIA, and the proportion of secreted insulin in the islet insulin content was shown in (A, right panel). The proinsulin and insulin contents of L1 and Wt islets were determined by insulin antisera alone on the same blot membrane, normalized by tubulin, and shown in (B). Data in (A and B) were shown as mean ± SD. n = 6.
(TIF)
