# Supplementary figures and images for: β-Cells with Relative Low HIMP1 Overexpression Levels in a Transgenic Mouse Line Enhance Basal Insulin Production and Hypoxia/Hypoglycemia Tolerance
Source: PLoS One. 2012 Mar 21;7(3):e34126. doi: 10.1371/journal.pone.0034126 (PMC3309936; doi:10.1371/journal.pone.0034126)

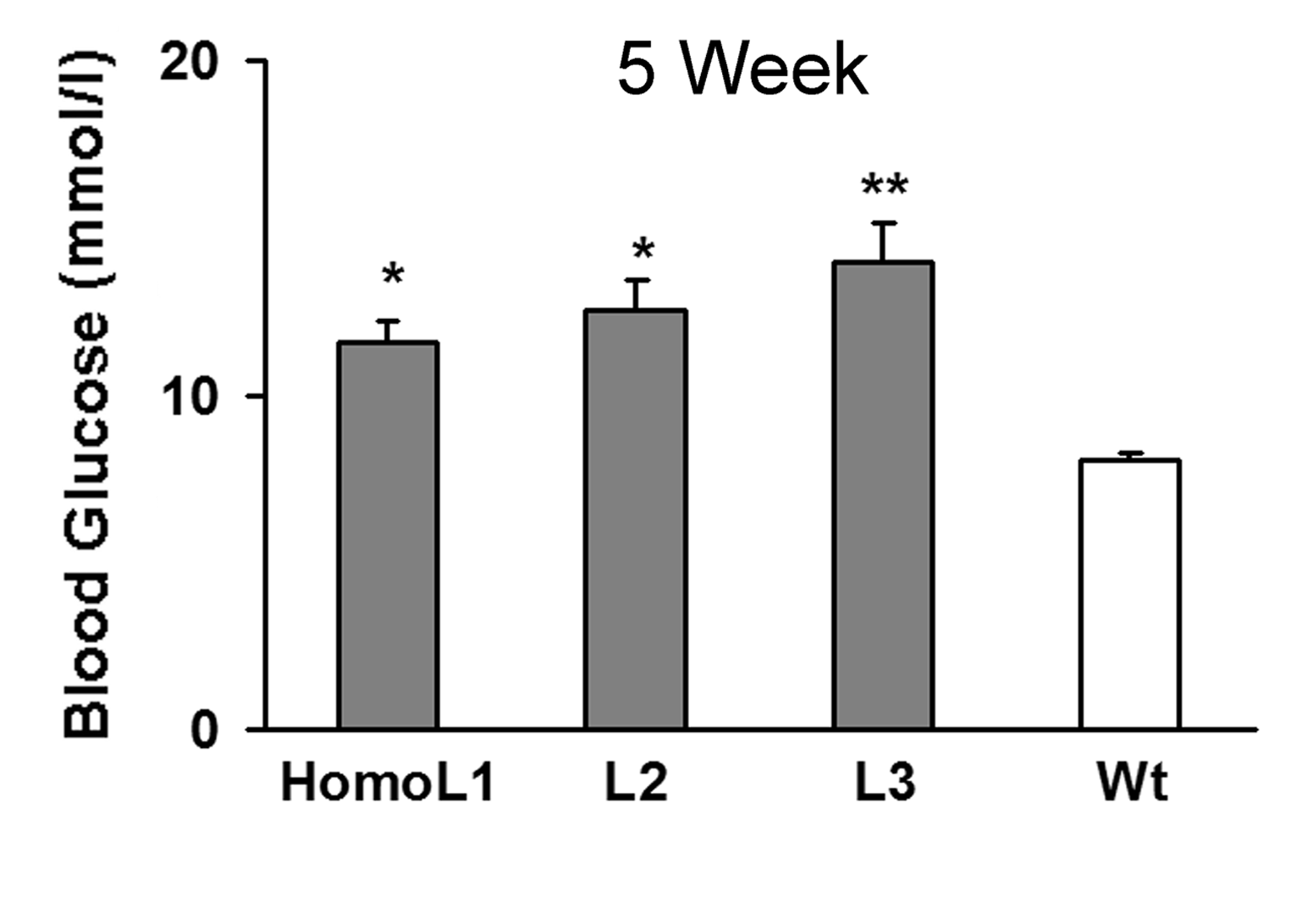

Supplement: Figure S1 — Blood glucose concentrations of 5-weeks-old L1 homozygotes and L2/3 heterozygotes. (TIF) [file pone.0034126.s001.tif]

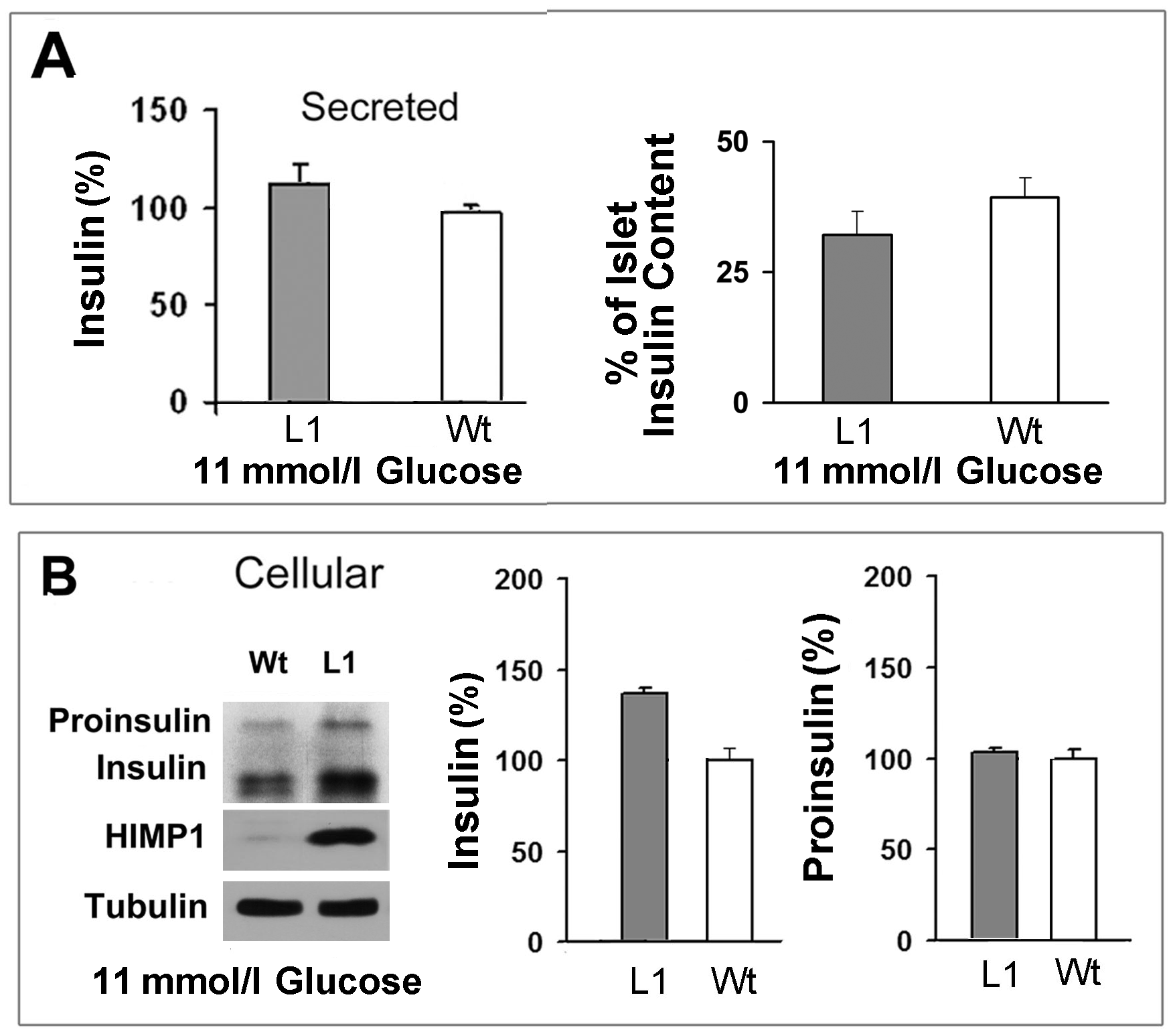

Supplement: Figure S2 — The (pro)insulin content and secreted insulin level of HIMP1-Tg-L1 and control islets after 15 h culture at the 11 mmol/l glucose condition. Islets isolated from 5-week-old heterozygous HIMP1-Tg-L1 or wild-type (Wt) mice were cultured for 15 h at 2.5 mmol/l glucose conditions. Insulin secreted during the 15 h culture was examined by RIA, and the proportion of secreted insulin in the islet insulin content was shown in (A, right panel). The proinsulin and insulin contents of L1 and Wt islets were determined by insulin antisera alone on the same blot membrane, normalized by tubulin, and shown in (B). Data in (A and B) were shown as mean ± SD. n = 6. (TIF) [file pone.0034126.s002.tif]
